# Process optimization of centrifugal dehydration–hydrocolloid pretreatments for quality preservation of frozen kimchi

**DOI:** 10.1038/s41598-026-48286-9

**Published:** 2026-04-14

**Authors:** Yun-Jeong Choi, Hee Eun Kim, Min Jung Lee, Minji Kim, Sung Jin Park, Ji Young Choi, Miran Kang, Sung Hee Park, Mi-Ai Lee

**Affiliations:** 1 Sustainable Distribution Research Group, World Institute of Kimchi, Gwangju, Korea; 2 Department of Integrative Food, Bioscience and Biotechnology, Chonnam National University, Gwangju, 61186 Republic of Korea

**Keywords:** Frozen kimchi, Cryoprotectant, Hydrocolloid, Microstructure, Quality improvement, Biochemistry, Biotechnology, Chemistry

## Abstract

The growing global demand for kimchi has led to the adoption of frozen distribution as a strategy to extend shelf-life. However, freezing can cause quality deterioration through ice crystal-related structural damage, resulting in texture softening and reduced microbial viability. We evaluated pretreatment strategies to improve the quality stability of frozen kimchi, focusing on the combined use of centrifugal dehydration and formulation-based cryoprotectant/hydrocolloid additions. Five pretreatment approaches were tested, including centrifugal dehydration, glucose addition, and hydrocolloid incorporation using sodium carboxymethyl cellulose and xanthan gum. Centrifugal dehydration was the primary factor reducing moisture content and thawing loss and thereby improving texture retention during frozen storage. Glucose and hydrocolloid treatments exhibited limited, index-dependent additional effects, with modest differences observed in selected parameters such as reducing sugar (RS) levels, lactic acid bacteria (LAB) counts, and antioxidant-related indices (total phenolic content (TPC) and DPPH radical-scavenging activity (DPPH)). Principal component analysis (PCA) suggested that the RS levels, LAB counts, and texture variables were associated with overall quality variation among treatments during frozen storage. Overall, centrifugal dehydration serves as an effective baseline pretreatment for frozen kimchi, while glucose/hydrocolloid additions may offer incremental stabilization in specific quality indices, warranting further validation under longer storage durations and diverse distribution conditions.

**Supplementary Information:**

The online version contains supplementary material available at 10.1038/s41598-026-48286-9.

## Introduction

Kimchi is a representative fermented food of Korea and is recognized as a health food worldwide because of its abundant lactic acid bacteria (LAB) and functional ingredients^1^. Recently, kimchi exports have been increasing, but quality deterioration remains a problem owing to continuous fermentation during distribution and storage. This deterioration manifests as production of acids, generation of carbon dioxide, and changes in texture and physical properties^2,3^. To solve this problem, various methods have been studied, including preservative treatment^4^, high-pressure treatment^5^, ultra-cooling^6,7^, and microbial control technologies^8^. However, these methods have limitations in their commercial application^9^.

Accordingly, the demand for frozen kimchi is increasing as an alternative for long-term distribution. However, the freezing process is known to cause quality deterioration, such as cell wall destruction, increased thawing loss (TL), decreased hardness, and reduced LAB count due to the formation of large ice crystals in the tissue^10,11^. Various pretreatment technologies have been proposed to solve these problems. For example, the cryoprotective effects of sugars such as glucose, sucrose, and trehalose have been reported^12,13^. Osmotic dehydration, a process that lowers the freezing point and reduces tissue damage by removing free water and increasing solid content, is one such technology^14^. In addition, hydrocolloids (e.g., sodium carboxymethyl cellulose (CMC), and xanthan gum) have been reported to contribute to structural stabilization and improved texture after thawing by enhancing viscosity and water retention capacity^15,16^. Mechanistically, sugars may modulate freezing-point depression and freeze concentration, whereas hydrocolloids can bind to water and reduce water mobility during thawing; however, the extent to which these mechanisms translate into measurable benefits in complex kimchi matrices remains poorly established.

The dehydration process plays a key role in improving the quality of frozen kimchi^17^. Centrifugal dehydration, in particular, has recently been reported as an effective method for reducing moisture and stabilizing quality in a short period, especially when compared to conventional dehydration methods^18,19^. However, it is essential to set appropriate conditions, as excessive centrifugal force can cause hardness, degradation, and cell wall damage^20^. Additionally, most studies are limited to frozen vegetables or kimchi raw materials. There is a notable lack of comprehensive studies evaluating the effects of centrifugal dehydration and its combined pretreatment with glucose and hydrocolloid on frozen kimchi. Thus, evidence remains limited for an application-oriented evaluation of an integrated pretreatment (centrifugal dehydration with glucose and a CMC/xanthan system) in a seasoned kimchi matrix, linking TL/texture with microbial and functional-related indices during frozen storage.

Therefore, in this study, we evaluated the effects of combined pretreatment using centrifugal dehydration, glucose (cryoprotectant), and hydrocolloids (CMC and xanthan gum) to improve TL and the physical properties of frozen kimchi. We aimed to analyze changes in the physicochemical, functional, and microbiological quality characteristics of frozen kimchi and to propose the optimal pretreatment method. We hypothesized that centrifugal dehydration is the primary driver of freeze–thaw stability by reducing freezable water content and TL, while glucose and CMC/xanthan gum would provide modest, index-dependent additional stabilization by modulating water mobility and limiting structural disruption and solute loss during thawing. To test this hypothesis, we compared pretreatments using centrifugal dehydration alone and in combination with glucose and hydrocolloids, and quantified TL, texture parameters, microbial counts, and functional-related indices during 3 months of frozen storage.

## Results and discussion

### Thawing loss and hardness of frozen kimchi

**Fig. 1:**
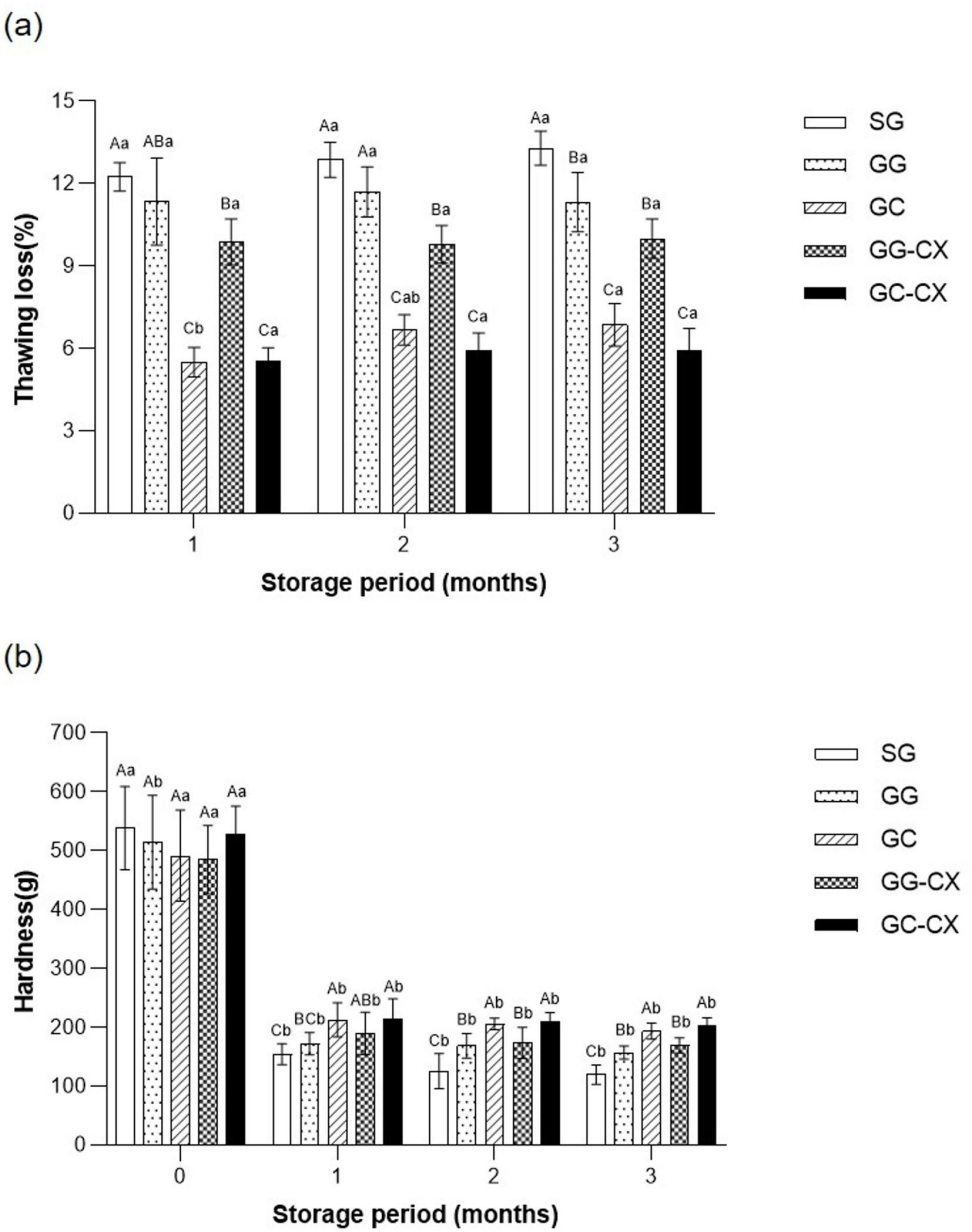
Effect of different dehydration and hydrocolloid treatments on the thawing loss **(a)** and hardness **(b)** of frozen kimchi during storage. SG, general dehydration with salt; GG, general dehydration with glucose; GC, centrifugal dehydration with glucose; GG-CX, general dehydration with glucose and added mixture of CMC and xanthan gum; GC-CX, centrifugal dehydration with glucose and mixture of CMC and xanthan gum. Values represent the mean ± SD (*n* = 3). Statistical differences were determined by one-way ANOVA followed by Duncan’s multiple range test (*p* < 0.05). Different uppercase letters within the same storage month indicate significant differences among treatments, and different lowercase letters within the same treatment indicate significant differences among storage months.

Figure 1 shows the TL and hardness of frozen kimchi under various pretreatment conditions. The TL of kimchi gradually increased during the 3-month frozen storage period, with no significant differences among the treatment groups, except for the GC (centrifugal dehydration with glucose) group (Fig. 1a). Significant differences were noted among the treatment groups after 1 month of frozen storage (*p* < 0.05). The GC-CX (centrifugal dehydration with glucose and a mixture of CMC and xanthan gum) group demonstrated the lowest TL (5.87% ± 0.49%), followed by the GC (6.59% ± 0.52%) and GG-CX (general dehydration with glucose and a mixture of CMC and xanthan gum) groups (7.23% ± 0.45%), with the SG (general dehydration with salt) group showing the highest value (9.15% ± 0.67%). These results suggest that centrifugal dehydration and hydrocolloid addition effectively minimized moisture loss during thawing. The superior performance of GC and GC-CX aligns with previous research findings, indicating that centrifugal dehydration reduces free water content before freezing, resulting in smaller ice crystals and less cellular damage during thawing^13,21^. Hydrocolloid incorporation (GG-CX and GC-CX) may further reduce drip formation by increasing water-binding capacity and reducing water mobility during thawing; however, water mobility and ice-crystal morphology were not directly measured in this study and, therefore, the proposed mechanisms should be interpreted as plausible mechanisms^16^.

The hardness analysis results revealed a general decrease during storage across all treatments (Fig. 1b), a typical phenomenon in frozen vegetables caused by cell damage from ice crystals^10,22^. Before freezing, all samples exhibited a hardness value of approximately 500 g. From the first month of freezing, the GC-CX group maintained the highest hardness (154.61 ± 7.43 g), followed by the GC group (148.33 ± 5.22 g), with the SG group showing the lowest hardness (123.40 ± 4.18 g). These results suggest that centrifugal dehydration not only reduces TL but also helps preserve the structural integrity of frozen kimchi by limiting free water and preventing large ice crystal formation^23^. The improved texture observed in the GC-CX group may be further enhanced by hydrocolloids, which help stabilize cell walls and limit excessive moisture loss during freezing and thawing^16,24^. The inverse relationship between TL and firmness underscores that moisture control is crucial for preserving the texture of frozen kimchi. Overall, these results support centrifugal dehydration as an effective baseline pretreatment for reducing TL and improving texture stability, with hydrocolloid addition providing limited, time point-dependent additional benefits.

### Microstructure of frozen kimchi

**Fig. 2:**
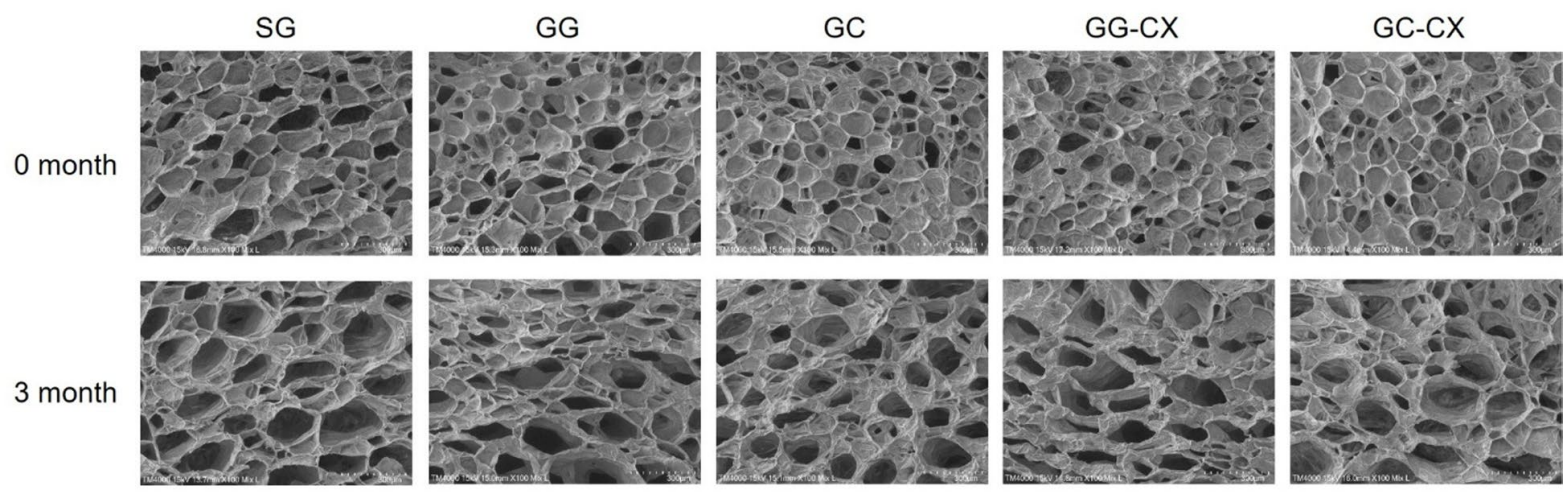
Effects of different dehydration and hydrocolloid treatments on the microstructure of frozen kimchi after three months of storage. Representative pores/intercellular spaces are indicated by arrows/circles (see annotations), and scale bars are shown in each panel. Annotations highlight representative void features for qualitative comparison (not a full quantitative pore-size distribution). SG, general dehydration with salt; GG, general dehydration with glucose; GC, centrifugal dehydration with glucose; GG-CX, general dehydration with glucose and mixture of CMC and xanthan gum; GC-CX, centrifugal dehydration with glucose and mixture of CMC and xanthan gum.

The microstructure of frozen foods significantly influences their physicochemical properties and overall quality. Freezing can cause cellular damage, leading to structural deformation, loss of texture, and nutrient leakage^25^. The microstructural characteristics of kimchi after 3 months of frozen storage were visualized using scanning electron microscopy (SEM) (Fig. 2). Significant structural differences were observed depending on the dehydration method and hydrocolloid treatment. The SG and GG groups exhibited enlarged intercellular spaces and irregular pore structures, along with severe cell wall collapse, indicating substantial damage caused by ice crystal formation during freezing. Such damage is commonly observed in frozen vegetables due to the formation of large intracellular ice crystals when moisture is insufficiently removed prior to freezing^13,15^.

In contrast, samples subjected to centrifugal dehydration (GC and GC-CX) demonstrated relatively well-preserved cell structures with small pore sizes and minimal tissue destruction. This finding suggests that centrifugal dehydration effectively reduces free water content before freezing, inhibiting excessive ice crystal growth and maintaining tissue integrity^19,23^. The GC-CX group, in particular, exhibited the most intact microstructure among all treatment groups, characterized by dense cell walls and minimal intercellular gaps. The improved microstructure in hydrocolloid-treated samples (GG-CX and GC-CX) can be attributed to the stabilizing effect of CMC and xanthan gum. These hydrocolloids are known to reduce water mobility during freezing and strengthen cell wall structure, thereby protecting against mechanical stress induced by ice formation^16,26^. Notably, the SEM observations qualitatively aligned with the macroscopic quality indices, as treatments showing reduced void expansion and less collapse (particularly GC and GC-CX) also exhibited lower TL and higher hardness. These results support centrifugal dehydration as the primary contributor to structural preservation, while hydrocolloid addition may provide incremental stabilization in selected conditions.

### Chemical properties of frozen kimchi

The pH of the kimchi samples decreased in all treatment groups during 3 months of storage (Table 1), with an initial range of 5.56–5.61. The lowest pH was observed in the GG-CX group (5.46 ± 0.01) and the highest in the GC group (5.55 ± 0.01). The decrease in pH was greater in the hydrocolloid-added groups (GG-CX and GC-CX), which is interpreted a result of the hydrocolloid creating a favorable environment for LAB^8^. Conversely, the pH stability of GC and GC-CX can be attributed to the moisture loss from dehydration, which suppresses microbial activity^19^. This finding is consistent with the findings of previous studies deciphering that the pH change in frozen vegetables was not significant^9,27^.

**Table 1:** Effects of hydrocolloids and dehydration on the chemical properties of frozen kimchi during storage.

Properties	Samples^1)^	Storage period (months)			
		0	1	2	3
pH	SG	5.61 ± 0.01^Ba^	5.61 ± 0.01^Aba^	5.56 ± 0.02^ABb^	5.50 ± 0.01^Bc^
	GG	5.60 ± 0.01^Ba^	5.61 ± 0.02^Aba^	5.56 ± 0.02^Bb^	5.50 ± 0.01^Bc^
	GC	5.59 ± 0.01^Aa^	5.63 ± 0.02^Ab^	5.59 ± 0.02^Ac^	5.55 ± 0.01^Ad^
	GG-CX	5.56 ± 0.01^Ca^	5.57 ± 0.01^Ca^	5.51 ± 0.02^Cb^	5.46 ± 0.01^Cc^
	GC-CX	5.61 ± 0.01^Ba^	5.60 ± 0.02^Ba^	5.56 ± 0.02^Bb^	5.50 ± 0.01^Bc^
Salinity (%)	SG	1.94 ± 0.01^Cb^	1.98 ± 0.02^Aa^	1.97 ± 0.02^Aa^	1.90 ± 0.01^Bc^
	GG	1.97 ± 0.01^Ba^	1.92 ± 0.02^Bb^	1.99 ± 0.03^Aa^	1.98 ± 0.01^Aa^
	GC	1.87 ± 0.01^Eb^	1.88 ± 0.01^Cab^	1.90 ± 0.02^Ca^	1.88 ± 0.01^Cab^
	GG-CX	2.00 ± 0.01^Aab^	1.99 ± 0.02^Aab^	2.01 ± 0.01^Aa^	1.99 ± 0.01^Ab^
	GC-CX	1.89 ± 0.01^Db^	1.87 ± 0.02^Cc^	1.94 ± 0.01^Ba^	1.82 ± 0.01^Dd^
Reducing sugar (mg/mL)	SG	36.29 ± 1.20^Cd^	43.33 ± 0.56^Cc^	45.94 ± 0.63^Cb^	48.51 ± 0.38^Ca^
	GG	45.98 ± 0.76^Bc^	51.21 ± 2.53^Bb^	58.15 ± 0.27^Ba^	59.75 ± 0.67^Ba^
	GC	53.66 ± 0.89^Ac^	57.79 ± 1.51^Ab^	67.20 ± 0.92^Aa^	67.03 ± 0.51^Aa^
	GG-CX	47.30 ± 0.47^Bc^	52.02 ± 1.52^Bb^	58.07 ± 0.38^Ba^	59.26 ± 0.55^Ba^
	GC-CX	52.34 ± 0.24^Ac^	59.63 ± 0.50^Ab^	66.19 ± 0.63^Aa^	66.56 ± 0.42^Aa^
Moisture content (%)	SG	87.14 ± 0.26^Aa^	86.71 ± 0.27^Aa^	86.80 ± 0.23^Aa^	86.72 ± 0.11^Aa^
	GG	86.23 ± 0.29^Ba^	86.21 ± 0.20^Ba^	85.83 ± 0.11^Bb^	85.18 ± 0.14^Bc^
	GC	85.02 ± 0.08^Ca^	84.62 ± 0.30^Cb^	84.50 ± 0.04^Db^	84.31 ± 0.28^Cb^
	GG-CX	86.05 ± 0.10^Ba^	86.09 ± 0.04^Ba^	85.52 ± 0.11^Cb^	85.45 ± 0.05^Bb^
	GC-CX	84.66 ± 0.11^Da^	84.63 ± 0.10^Ca^	84.00 ± 0.19^Eb^	83.75 ± 0.19^Db^

^1)^ SG, general dehydration with salt; GG, general dehydration with glucose; GC, centrifugal dehydration with glucose; GG-CX, general dehydration with glucose and mixture of CMC and xanthan gum; GC-CX, centrifugal dehydration with glucose and mixture of CMC and xanthan gum.

^2)^ All values are expressed as mean ± SD of three independent batches (*n* = 3).

^3)^ Data were analyzed by one-way ANOVA followed by Duncan’s multiple range test (*p* < 0.05).

^4)^ Different uppercase letters within the same column indicate significant differences among treatment groups at the same storage month, and different lowercase letters within the same row indicate significant differences among storage months within the same treatment (*p* < 0.05).

Salinity was relatively low in the GC and GC-CX groups and remained stable without significant fluctuations during storage. This is likely due to the initial moisture and salt loss during centrifugal dehydration^19^. In general, hydrocolloids are known to contribute to increased salt concentration^16^, but in this study, the dehydration method had a greater effect on salinity.

The reducing sugar (RS) level increased in all treatment groups during the storage period (*p* < 0.05), with the SG group having the lowest content due to the absence of added glucose. Among the glucose-added groups, the GC and GC-CX groups showed higher RS level than the GG and GG-CX groups, suggesting that dehydration-related changes in water content and extractability contributed to higher measured RS level. However, a simple concentration effect alone is unlikely to explain the magnitude of increase in RS level when the changes in moisture content are minimal. Freeze–thaw-induced tissue disruption may increase the extractability of soluble carbohydrates, and endogenous enzymatic hydrolysis of polysaccharides occurring in localized unfrozen fractions and/or during thawing could also contribute to RS formation^28,29^. The addition of hydrocolloid is thought to have contributed to the preservation of RS by inhibiting moisture mobility^16^.

Moisture content showed a significant difference among the treatment groups, with the GC and GC-CX groups maintaining the lowest moisture content from the beginning of storage. The decrease in moisture content during storage was also the greatest in the GC-CX group, which can be interpreted as an effect of reduced moisture content through dehydration and TL. In particular, the GC-CX treatment seems to have positively contributed to the inhibition of TL through moisture content reduction and preservation of functional ingredients^19,28^.

### Microbial properties of frozen kimchi

The changes in total viable bacteria (TVB) and LAB counts of frozen kimchi are presented in Table 2. The initial TVB count ranged from 6.59 to 6.78 log CFU/mL, with the GC-CX group showing the highest value (6.78 ± 0.06 log CFU/mL) and the GG-CX group showing the lowest value (6.59 ± 0.05 log CFU/mL). After 3 months, there was no significant difference among the treatment groups. Although there was a slight decrease during the storage period, all treatment groups maintained relatively stable TVB counts, indicating no significant treatment effect on TVB counts. These results are consistent with those of previous studies showing that the total bacterial population, consisting of LAB and non-LAB species, is generally resilient to frozen storage conditions^11,28^.

**Table 2:** Effects of hydrocolloids and dehydration on the microbial properties of frozen kimchi during storage.

Properties	Samples^1)^	Storage period (months)			
		0	1	2	3
Total viable bacteria (log CFU/mL)	SG	6.67 ± 0.06^Ba^	6.64 ± 0.04^ABa^	6.63 ± 0.03^Aa^	6.58 ± 0.07^Aa^
	GG	6.62 ± 0.03^Ba^	6.65 ± 0.06^ABa^	6.61 ± 0.03^Aa^	6.60 ± 0.06^Aa^
	GC	6.68 ± 0.06^Ba^	6.69 ± 0.02^Aa^	6.60 ± 0.05^Ab^	6.70 ± 0.04^Aa^
	GG-CX	6.59 ± 0.05^Ba^	6.57 ± 0.02^Ba^	6.63 ± 0.02^Aa^	6.63 ± 0.06^Aa^
	GC-CX	6.78 ± 0.06^Aa^	6.61 ± 0.09^ABb^	6.66 ± 0.04^Aab^	6.63 ± 0.10 ^Ab^
Lactic acid bacteria (log CFU/mL)	SG	6.28 ± 0.06^ABa^	5.76 ± 0.03^Cb^	5.63 ± 0.08^Cc^	5.53 ± 0.07 ^Cc^
	GG	6.34 ± 0.03^ABa^	5.75 ± 0.03^Cb^	5.67 ± 0.01^BCc^	5.64 ± 0.02^Bc^
	GC	6.36 ± 0.05^Aa^	6.00 ± 0.02^Ab^	5.75 ± 0.02^Bc^	5.72 ± 0.05^ABc^
	GG-CX	6.25 ± 0.04^Ba^	5.81 ± 0.03^Bb^	5.70 ± 0.06^BCc^	5.66 ± 0.04^Bc^
	GC-CX	6.33 ± 0.05^ABa^	6.01 ± 0.03^Ab^	5.83 ± 0.03^Ac^	5.79 ± 0.02^Ac^

^1)^ SG, general dehydration with salt; GG, general dehydration with glucose; GC, centrifugal dehydration with glucose; GG-CX, general dehydration with glucose and mixture of CMC and xanthan gum; GC-CX, centrifugal dehydration with glucose and mixture of CMC and xanthan gum.

^2)^ All values are expressed as mean ± SD of three independent batches (*n* = 3).

^3)^ Data were analyzed by one-way ANOVA followed by Duncan’s multiple range test (*p* < 0.05).

^4)^ Different uppercase letters within the same column indicate significant differences among treatment groups at the same storage month, and different lowercase letters within the same row indicate significant differences among storage months within the same treatment (*p* < 0.05).

On the contrary, the LAB count decreased significantly during frozen storage, consistent with the known sensitivity of LAB to freeze–thaw stress. The initial LAB counts ranged from 6.25 ± 0.04 to 6.36 ± 0.05 log CFU/mL. After 3 months, the overall LAB counts decreased, with the GC-CX group showing the highest LAB viability (5.79 ± 0.02 log CFU/mL), whereas the SG group had to the lowest level (5.53 ± 0.07 log CFU/mL) (*p* < 0.05). The relatively higher LAB retention in the GC and GC-CX groups may be associated with reduced free water content and improved matrix integrity following centrifugal dehydration, which could mitigate freeze–thaw injury to cells^15,19^. Additionally, the hydrocolloid-treated groups (GG-CX and GC-CX) maintained higher LAB counts than the non-hydrocolloid control groups (SG, GG, and GC), which may reflect hydrocolloid-mediated modulation of water mobility in the matrix^23,30^. However, specific protective mechanisms were not directly measured in this study. Importantly, maintaining LAB viability at an industrially relevant threshold (e.g., ≥10^6^ CFU/g) is a key practical consideration for frozen kimchi; therefore, longer-term storage studies and evaluation of prebiotic/protective formulations are warranted to confirm viability targets and post-thaw fermentation performance.

### Bioactive compounds of frozen kimchi

DPPH is a stable free radical commonly employed to evaluate antioxidant activity by reacting with sample extracts^31^. As DPPH activity reflects the scavenging capacity toward a specific radical under defined extraction/solvent conditions, it serves as a useful but partial index of antioxidant potential; therefore, the DPPH activity assay findings should be complemented by other indicators and discussed by considering methodological limitations^32^. A key contributor to DPPH activity is total phenolic content (TPC), which comprises various plant-derived secondary metabolites and plays a crucial role in antioxidant and antimicrobial properties by interacting with proteins and other macromolecules^33^. In addition, lactic acid bacteria (LAB) can modulate antioxidant potential by releasing bound phenolics and biotransforming phenolic compounds via microbial enzymes during fermentation, which may alter DPPH/TPC assay outcomes^34^. However, cell damage during freezing and storage can result in the release and degradation of antioxidant compounds through chemical and enzymatic oxidation, leading to lower antioxidant activity than that of the fresh product^35^. Notably, freeze–thaw injury may simultaneously increase extractability (by disrupting tissues) while accelerating oxidation and loss of labile compounds; thus, the net change in DPPH activity and TPC reflects the balance between enhanced extract release and oxidative degradation.

**Fig. 3:**
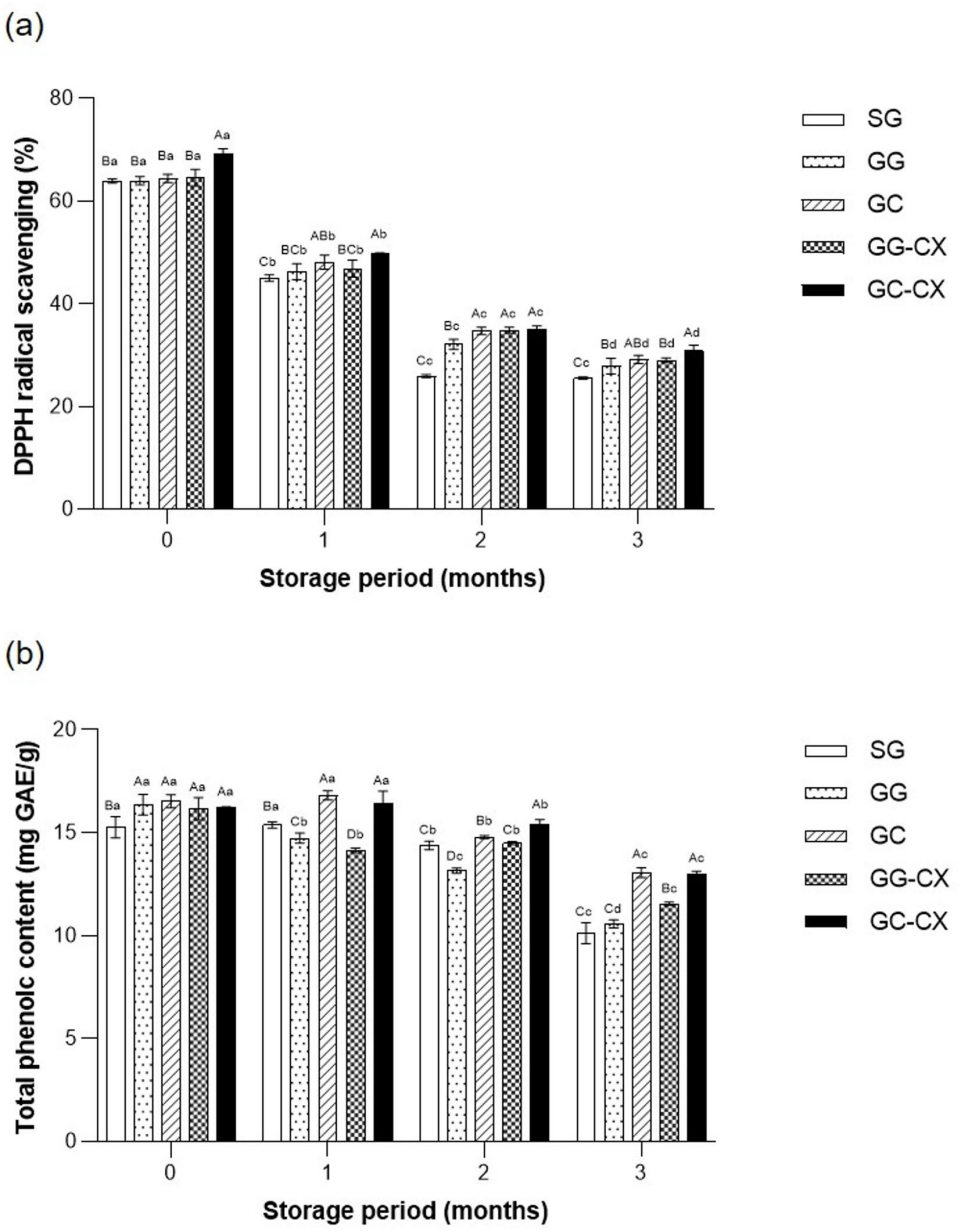
Changes in antioxidant activity **(a)** and total phenolic content **(b)** of frozen kimchi during storage under different dehydration and hydrocolloid treatments. SG, general dehydration with salt; GG, general dehydration with glucose; GC, centrifugal dehydration with glucose; GG-CX, general dehydration with glucose and mixture of CMC and xanthan gum; GC-CX, centrifugal dehydration with glucose and mixture of CMC and xanthan gum. Values represent the mean ± SD (*n* = 3). Statistical differences were determined by one-way ANOVA followed by Duncan’s multiple range test (*p* < 0.05). Different uppercase letters within the same storage month indicate significant differences among treatments, and different lowercase letters within the same treatment indicate significant differences among storage months.

The antioxidant activity (DPPH radical scavenging activity) and TPC of frozen kimchi significantly decreased across all treatment groups during the 3-month frozen storage period (Fig. 3a, b). Before freezing, the DPPH activity ranged from 63.47% ± 2.15% (SG) to 71.19% ± 1.48% (GC-CX). After 1 month of frozen storage, the DPPH activity declined in all groups, with the GC-CX group maintaining the highest activity (65.06% ± 1.41%) and the SG group showing the lowest activity (56.08% ± 2.26%). Similarly, TPC significantly decreased during frozen storage (*p* < 0.05). The initial TPC ranged from 36.12 ± 0.92 mg GAE/100 g (SG) to 42.30 ± 1.04 mg GAE/100 g (GC-CX), with the final values after 3 months being 32.19 ± 0.65 mg GAE/100 g (SG) and 38.84 ± 1.17 mg GAE/100 g (GC-CX).

The above-mentioned findings align with those of previous studies demonstrating that freezing and thawing can induce oxidative stress and cell damage, leading to a decrease in antioxidant components^36^. However, samples treated with centrifugal dehydration and hydrocolloids (GC-CX and GG-CX) retained significantly higher DPPH activity and TPC than those without hydrocolloids (*p* < 0.05). This preservation can be attributed to two primary factors: first, reduced water content due to centrifugal dehydration minimized cell disruption caused by ice crystals, thereby limiting the loss of soluble phenolic compounds^13,37^; second, the water-binding capacity of CMC and xanthan gum likely stabilized cell structure and protected biologically active compounds during freezing^35^. Additionally, the higher RS level observed in the GC-CX and GG-CX groups may have contributed to improved antioxidant activity. RS can act as cryoprotectants, stabilizing cell structure and reducing oxidative damage^15^. The synergistic effect of dehydration and hydrocolloids suggests that their combined application effectively preserves antioxidant properties during frozen storage. Nevertheless, as DPPH activity captures only one aspect of antioxidant behavior, future work incorporating complementary assays (e.g., ABTS) and oxidation markers would strengthen functional interpretation and enable more comprehensive antioxidant profiling.

### Correlation analysis of quality parameters in frozen kimchi

**Fig. 4:**
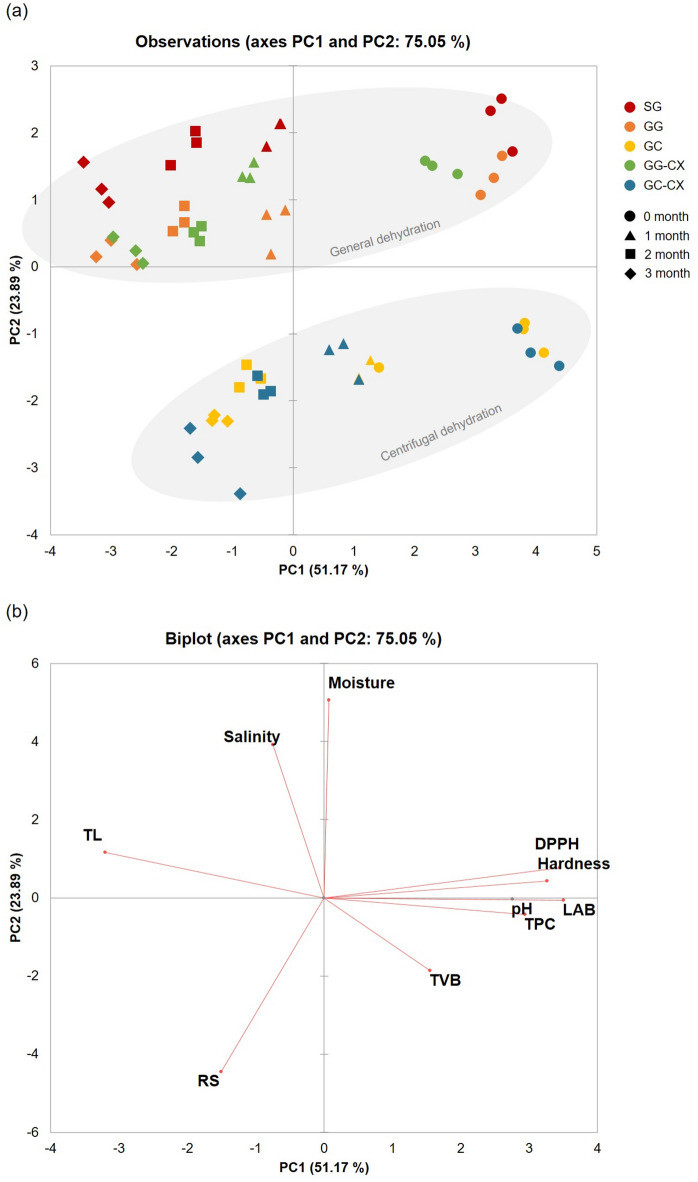
Principal component analysis (PCA) biplot illustrating the quality-related variables of frozen kimchi subjected to different pretreatments. PC1 and PC2 explained 51.17% and 23.89% of the variance, respectively (cumulative 75.05%). DPPH, DPPH radical scavenging activity; LAB, lactic acid bacteria; RS, reducing sugar; TL, thawing loss; TPC, total phenolic content; TVB, total viable bacteria.

In this study, principal component analysis (PCA) and correlation analysis were performed to identify the relationships among the physicochemical, functional, and microbiological quality characteristics of frozen kimchi and to evaluate the differences in these characteristics among the treatment groups. The results of the PCA (Fig. 4) revealed that PC1 (51.17%) and PC2 (23.89%) explained a total cumulative variance of 75.05%, revealing a clear distinction among the treatment groups based on the dehydration method and cryoprotectant application. The GC and GC-CX groups were located in the negative direction of PC1 and showed a high correlation with major quality factors such as RS, TVB count, and TPC. These PCA patterns suggest that variation among the treatment groups is largely structured along a “water status/structural integrity” axis, in which lower moisture content/TL tends to align with higher hardness and higher functional-related indices; however, the PCA reveals multivariate associations and does not establish causality. On the contrary, the SG group had high moisture content and TL rate, but low RS level and functional indices, indicating a clear decline in quality.

**Fig. 5:**
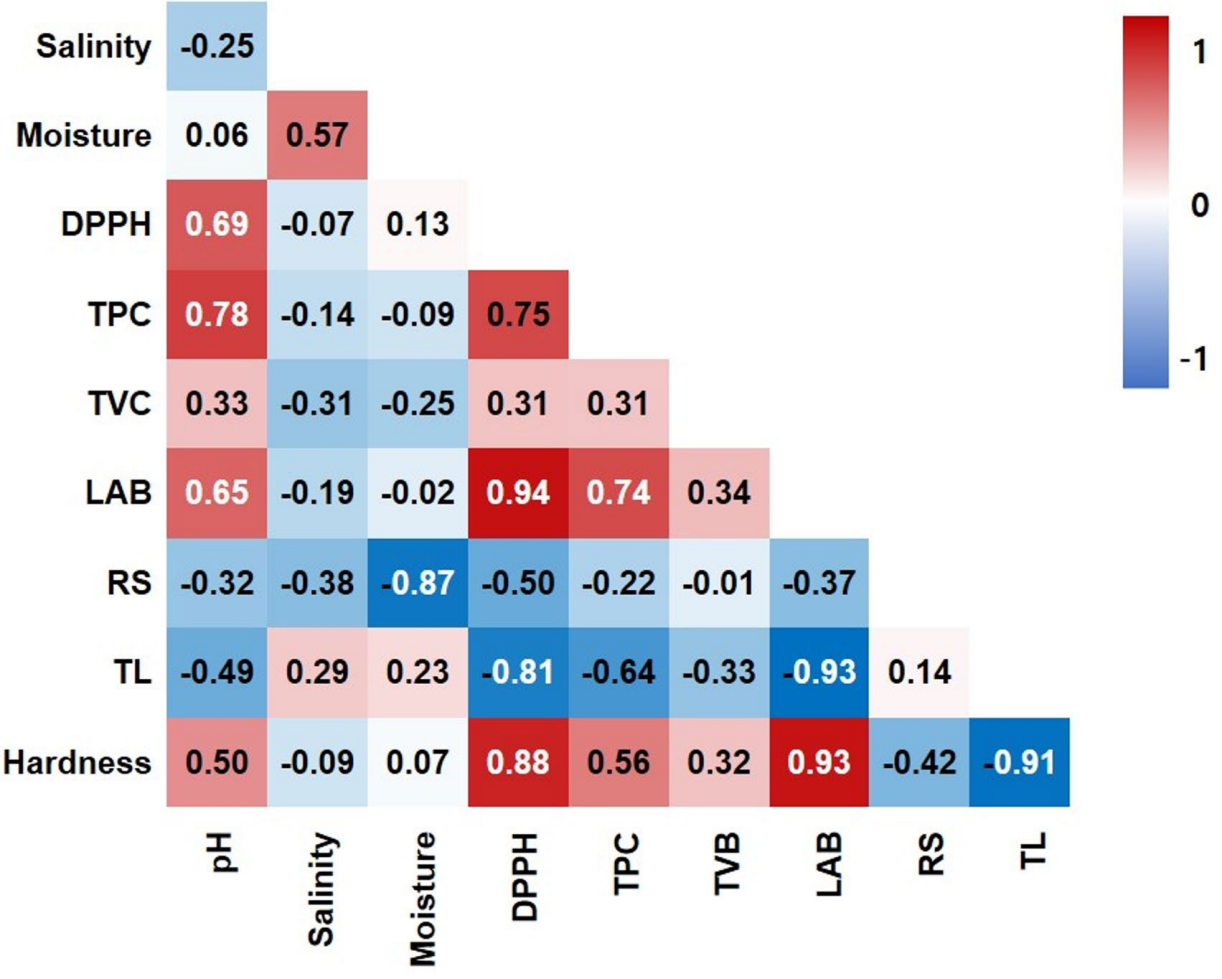
Correlation heatmap of physicochemical, microbial, and functional quality parameters in frozen kimchi. Heatmap shows Pearson correlation coefficients (r) among variables. DPPH, DPPH radical scavenging activity; LAB, lactic acid bacteria; RS, reducing sugar; TL, thawing loss; TPC, total phenolic content; TVB, total viable bacteria.

In the correlation analysis results (Fig. 5), hardness showed a significant positive correlation with DPPH activity (*r* = 0.88), TPC (*r* = 0.56), and LAB count (*r* = 0.93), while TL showed a strong negative correlation with hardness (*r* = -0.91), TPC (*r* = -0.64), and DPPH activity (*r* = -0.81). The high positive correlation among hardness, DPPH activity, TPC, and LAB count and their negative relationship with TL were consistent with the direction of the PCA loading vector, which more clearly explained the difference in quality characteristics among the treatment groups. Collectively, these relationships imply that moisture-related drip formation (TL) and structural collapse (hardness loss) are likely primary drivers of overall quality deterioration during frozen storage, and that antioxidant-related indices and LAB counts tend to co-vary with these physical changes. The findings suggest that the combination of glucose addition and centrifugal dehydration contributed to tissue stabilization and functional component preservation by suppressing quality deterioration during freezing. This was achieved by lowering the freezing point and inhibiting ice crystal formation through a decrease in free water content and an increase in solute concentration^12,24^. A plausible explanation is that reduced available/freezable water content and changes in solute distribution may mitigate freeze–thaw structural disruption; however, freezing-point depression, ice-crystal morphology, and water mobility were not directly quantified (e.g., using DSC or LF-NMR) in this study. Furthermore, the quality improvement effect of GC-CX treatment was also confirmed in comparison with that of GC treatment. This is likely due to the protective effect of CMC, which further contributed to the property improvement effect of centrifugal dehydration. These results are consistent with the findings of previous studies reporting that the main factors for quality deterioration during storage of frozen kimchi are moisture content and TL^10,11^. They also align with findings that the combined use of osmosis and centrifugal dehydration is effective in tissue protection and functional component preservation^14,37^. Therefore, GC-CX can be considered an application-relevant pretreatment option for improving TL/texture stability and maintaining selected microbial and functional-related indices of frozen kimchi under the tested conditions. From a practical perspective, this study provides an application-oriented benchmark of an integrated pretreatment in a seasoned kimchi matrix, showing that centrifugal dehydration is the primary contributor to TL/texture stability, while glucose/hydrocolloid additions yield limited, index-dependent incremental effects. Key limitations include the short storage period (3 months), single cultivar, and the lack of direct measurements of water mobility/thermal transitions (LF-NMR/DSC) and post-thaw LAB activity, which should be addressed in future studies.

## Conclusion

In this research, we examined the effects of combined pretreatments involving centrifugal dehydration, glucose addition, and hydrocolloid application (CMC and xanthan gum) on the quality of frozen kimchi. Centrifugal dehydration helped to preserve the textural properties of kimchi throughout frozen storage by reducing moisture content and minimizing TL. Across the tested conditions, centrifugal dehydration was the primary driver of TL reduction and texture retention. The combined treatment of glucose addition and hydrocolloid application showed limited, index-dependent additional retention of hardness, LAB count, TPC, and DPPH radical scavenging activity in frozen kimchi. These findings indicate potential incremental stabilization of selected function-related indices and microbiological viability, rather than broad improvements across all quality attributes. Through multivariate analysis (PCA and correlation analysis), RS level, LAB count, and hardness were identified as key factors associated with quality variation among treatments during frozen storage. In particular, the GC-CX treatment group generally exhibited favorable performance across multiple indices within the present experimental settings. Therefore, a pretreatment method that combines centrifugal dehydration with glucose and a hydrocolloid complex may serve as an effective baseline strategy (centrifugal dehydration) with incremental benefits from formulation additions for addressing TL and texture deterioration in frozen kimchi.

This study has some limitations. It was conducted using a single kimchi cabbage cultivar and a 3-month frozen storage period. Because this relatively short storage duration may limit detection of smaller formulation effects, longer-term validation (≥ 6–12 months) under distribution-relevant conditions is warranted. Industrial-scale validation and long-term distribution conditions were not examined and should be addressed in future research. In addition, key translation factors—including scalability/throughput and energy consumption of centrifugal dehydration, regulatory acceptance of food-grade glucose/CMC/xanthan gum, and potential sensory effects were not evaluated and should be assessed in industrial validation studies. Future studies should evaluate longer storage durations, batch-to-batch variability, and post-thaw fermentation performance to confirm robustness and practical relevance. Nevertheless, our approach provides a useful basis for the further development and industrial application of frozen kimchi products.

## Materials and methods

### Experimental materials

Kimchi cabbage and garlic were purchased from a western agricultural and fishery market in Gwangju, Republic of Korea. Other ingredients, including red pepper powder (Hansaeng, Seocheon-gun, Republic of Korea), fermented anchovy sauce (Chung Jung One, Daesang Corp., Seoul, Republic of Korea), sugar (Beksul, CJ CheilJedang Corp., Seoul, Republic of Korea), and purified salt (Hanju Salt, Ulsan, Republic of Korea), were sourced from an online market. Glucose (ESfood, Gunpo, Republic of Korea), CMC (Changshu Wealthy Science and Technology Co., Ltd, Changshu, China), and xanthan gum (ESfood, Gunpo, Republic of Korea) were also purchased from an online market. All experimental analyses were performed using first-grade analytical reagents obtained from Daejung (Gyeonggi-do, Republic of Korea).

### Preparation of frozen kimchi

The prepared kimchi cabbage was cut into 30 mm × 30 mm pieces and then salted. The salting process varied between the groups: Group S was salted with 5% refined salt (w/w), while Group G was salted with 5% refined salt and 5% glucose (w/w) for 1 h. After salting, the kimchi cabbage was washed three times with tap water, divided into halves, and residual brine was removed using two dehydration methods: general dehydration (G) and centrifugal dehydration (C). The G sample was drained for 2 h, whereas the C sample was treated at 225 × *g* for 5 min using a spin dryer (SDM-T77H; Shinil Electronics, Cheonan, Republic of Korea).

Kimchi seasoning was prepared by combining garlic (12.5%, w/w), red pepper powder (17.5%, w/w), anchovy sauce (15.0%, w/w), sugar (5.0%, w/w), and water (50.0%, w/w). For the CX treatment group, the water fraction (50.0%, w/w) was replaced with a hydrocolloid dispersion containing CMC (0.5%, w/w), xanthan gum (0.05%, w/w), and water (49.45%, w/w), resulting in the same total seasoning mass as that of the hydrocolloids incorporated. The concentrations of CMC (0.5%, w/w) and xanthan gum (0.05%, w/w) were selected based on preliminary screening to achieve measurable water-binding/coating effects without excessive thickening that could hinder mixing, uniform coverage, or sensory acceptability; these levels are within ranges commonly reported for texture/moisture stabilization in plant-based food matrices. The seasoning was added to the salted cabbage at a ratio of 80:20 (w/w; salted kimchi cabbage: seasoning) and mixed thoroughly.

Each kimchi sample (300 g), prepared using its respective treatment method, was individually packaged in polyethylene film (200 mm × 250 mm × 0.1 mm) and vacuum-packed using a packaging machine (Airzero AZC-070; INTRISE, Ansan, Republic of Korea). All samples were frozen at − 40 °C for 24 h in a freezer (QF-700; Seojin Freezer Co., Ltd., Goyang, Republic of Korea) and then stored at − 20 °C for 3 months. Day 0 was defined as immediately after seasoning and vacuum packaging, prior to freezing. For each sampling time (0, 1, 2, and 3 months), separate vacuum-packed units were used.

Frozen kimchi was thawed in running water at 18 ± 1.0 °C under a constant flow rate and temperature was monitored using a temperature sensor (CENTER-309; Center Technology Corp., Taipei, Taiwan) placed at the sample core. Thawing was considered complete when the core temperature reached 4 °C. After thawing, samples were gently drained for a fixed period (10 min) and analyzed immediately to minimize post-thaw fermentation and compositional changes.

### Thawing Loss

To calculate TL, the initial weight of the kimchi sample was measured and recorded as W_1_. After completing the freeze–thaw cycles, the samples were gravity-drained for 1 h and reweighed, with the new weight recorded as W_2_. Each measurement was performed in triplicate. TL, expressed as percentage of weight loss, was calculated using the following formula:

1 $$\:\mathrm{T}L\:\left({\%}\right)=\frac{{W}_{1}-{W}_{2}}{{W}_{1}}\times\:100$$

where, W_1_ is the initial weight (g) of kimchi before freezing and W_2_ is the final weight (g) of the thawed kimchi sample.

### Hardness measurements

The white parts of kimchi leaves have uniform textural properties, which enhance the accuracy of hardness measurements^38^. Ten white leaf samples were randomly selected from each treatment group. Hardness was measured using a CT3 texture analyzer (AMETEK Brookfield, MA, USA) equipped with a TA44 probe (diameter: 4 mm). The operating conditions of the texture analyzer were as follows: a trigger load of 5 g, deformation ratio of 40%, pre-test speed of 2.00 mm/s, test speed of 0.50 mm/s, and post-test speed of 2.00 mm/s.

### Microstructure analysis

SEM (TM4000Plus; Hitachi, Tokyo, Japan) was used to observe the microstructures of the samples after 3 months of frozen storage. The kimchi samples were first freeze-dried using a vacuum freeze drier (FD8508; ilSinBioBase Co., Ltd., Dongducheon, Republic of Korea). The lyophilized samples were precisely cut into pieces measuring 5 mm × 5 mm for microstructural observation and fixed onto the sample tray with double-sided tape. Cross-sectional images were acquired at a magnification of ×100 and accelerating voltage of 15 kV during scanning.

### pH and salinity

Thawed kimchi samples were homogenized using a blender (HP-1372; Philips, Guangdong, China). The homogenized mixture was then passed through a filter bag (Whirl-Pak 1195; Madison, WI, USA). The pH of the filtrate was measured at room temperature using a pH meter (TitroLine 5000; SI Analytics GmbH, Mainz, Germany). Salinity was measured using a salt meter (PAL-SALT; ATAGO, Tokyo, Japan). All measurements were performed in triplicate, and the instruments were rinsed with distilled water before each measurement.

### Reducing sugars

RS level was analyzed using the dinitrosalicylic acid (DNS) method^39^. The sample extract was diluted 25-fold with distilled water, and 1 mL of the diluted sample was mixed with 3 mL of DNS reagent. The mixture was then heated in a water bath at 100 °C for 5 min. After heating, the reaction mixture was rapidly cooled at 25 °C and further diluted with 16 mL of distilled water. The absorbance of the reaction mixture was measured at 550 nm using a microplate spectrophotometer (SPECTROstar Nano; BMG Labtech, Ortenberg, Germany). A standard calibration curve was prepared by reacting various concentrations of standard glucose (Sigma-Aldrich Co., St. Louis, MO, USA) with DNS reagent. Results were calculated based on a glucose standard curve.

### Moisture content

The treated samples were blended into a paste. A 1-g portion of the homogenized salted kimchi cabbage (SKC) was placed on the plate of an infrared moisture analyzer (MB45; OHAUS, NJ, USA) and heated to 105 °C. The measurement was considered complete when no weight change was detected for 60 s. The average moisture content was measured in triplicate for each group.

### Microbial properties

Kimchi samples were ground in a sterile blender and extracted using a sterile filter bag. To determine the number of TVB and LAB, the filtrate was diluted 10-fold with sterile saline (HAPS DW-9; HUKO FS Co., Ltd., Seoul, Republic of Korea) and vortexed for 30 s. The diluted solutions were then plated on ready-to-use aerobic count plates (3 M Petrifilm™, St. Paul, MN, USA) and total LAB count plates (3 M Petrifilm™). The plates were incubated for 2 days at 37 °C in an incubator (SI-600R; Jeio Tech, Seoul, Republic of Korea). Colony counts are expressed as log colony-forming units per milliliter (log CFU/mL).

### Bioactive compounds

#### DPPH radical scavenging activity

The DPPH radical scavenging activity of SKC was determined following a previously reported method^40^ with some modifications. A 60-µL aliquot of sample was mixed with 240 µL of a freshly prepared 0.1 mM DPPH radical solution. The mixture was then incubated in the dark at ambient temperature for 30 min. The absorbance (Abs) was measured at 517 nm using a spectrophotometer microplate reader (SPECTROstar Nano, BMG LABTECH). DPPH radical scavenging activity (%) was calculated using the following formula:

2 $${\text{DPPH activity }}\left( \% \right){\text{ }}={\text{ }}[({\text{ Ab}}{{\mathrm{s}}_{{\mathrm{control}}}}--{\mathrm{Ab}}{{\mathrm{s}}_{{\mathrm{sample}}}})/{\mathrm{Ab}}{{\mathrm{s}}_{{\mathrm{control}}}}] \times {\mathrm{1}}00$$

### Total phenolic content

TPC was measured following a previously reported method^33^. A 50-µL aliquot of sample was mixed with 50 µL of Folin–Ciocalteu reagent (Sigma-Aldrich, St. Louis, MO, USA), and then 150 µL of Na_2_CO_3_ solution was added. After 30 min reaction in the dark, the absorbance was measured at 765 nm using a spectrophotometer microplate reader (SPECTROstar Nano; BMG LABTECH). The TPC was calculated using a gallic acid standard curve.

### Statistical analysis

All measurements were performed using three independent batches (*n* = 3) per treatment. Data are presented as mean ± SD. Statistical analyses were performed using GraphPad Prism version 10 (GraphPad Software Inc., San Diego, CA, USA). PCA was carried out using XLSTAT-Base Perpetual version 19.4 (Addinsoft, New York, NY, USA) to visualize multivariate patterns and assess intrinsic variation across frozen storage period (0, 1, 2, and 3 months). For group comparisons, one-way analysis of variance (ANOVA) was conducted at each storage time point, followed by Duncan’s multiple range test to determine significant differences among treatments (*p* < 0.05). Within each treatment, temporal differences across storage months were evaluated using one-way ANOVA with Duncan’s multiple range test (*p* < 0.05).

## Supplementary Information

Below is the link to the electronic supplementary material.

Supplementary Material 1

## Data Availability

All data supporting the results of this study are available in the article. They can also be obtained from the corresponding author on reasonable request.
