# Supplementary material for: Process optimization of centrifugal dehydration–hydrocolloid pretreatments for quality preservation of frozen kimchi
Source: Sci Rep. 2026 Apr 14;16:17454. doi: 10.1038/s41598-026-48286-9 (PMC13237078; doi:10.1038/s41598-026-48286-9)
Supplement: Supplementary file 1 — Supplementary Material 1 [file 41598_2026_48286_MOESM1_ESM.docx]

**Supplementary materials**

**Process Optimization of Centrifugal Dehydration–Hydrocolloid Pretreatments for Quality Preservation of Frozen Kimchi**

Yun-Jeong Choi^1^, Hee Eun Kim^1,2^, Min Jung Lee^1^, Minji Kim^1^, Sung Jin Park^1^, Ji Young Choi^1^, Miran Kang^1^, Sung Hee Park^1^, Mi-Ai Lee^1,*^

*^1^ Sustainable Distribution Research Group, World Institute of Kimchi, Gwangju, Korea*

*^2^Department of Integrative Food, Bioscience and Biotechnology, Chonnam National University, Gwangju 61186, Republic of Korea*

***Corresponding author**

Mi-Ai Lee

Sustainable Distribution Research Group, World Institute of Kimchi, Gwangju, Korea

E-mail: [leemae@wikim.re.kr](mailto:leemae@wikim.re.kr)

**Supplementary tables for:**

Table S1. Pearson correlation coefficients (r) and significance levels (p-values) among physicochemical, microbial, and functional quality parameters of frozen kimchi. Variables include thawing loss (TL), hardness, lactic acid bacteria (LAB), DPPH radical scavenging activity (DPPH), total phenolic content (TPC), reducing sugars (RS), and total viable bacteria (TVB). Significant correlations are highlighted in bold (p < 0.05).

(a) Summary statistics

| Variable | Observations | Obs. with missing data | Obs. without missing data | Minimum | Maximum | Mean | Std. deviation |
| --- | --- | --- | --- | --- | --- | --- | --- |
| pH | 60 | 0 | 60 | 5.450 | 5.690 | 5.569 | 0.056 |
| salinity | 60 | 0 | 60 | 1.820 | 2.030 | 1.935 | 0.055 |
| moisture | 60 | 0 | 60 | 83.590 | 87.440 | 85.471 | 1.004 |
| DPPH | 60 | 0 | 60 | 25.324 | 74.101 | 43.525 | 15.066 |
| TPC | 60 | 0 | 60 | 9.698 | 17.079 | 14.430 | 1.962 |
| TVC | 60 | 0 | 60 | 6.505 | 6.845 | 6.638 | 0.065 |
| LAB | 60 | 0 | 60 | 5.447 | 6.415 | 5.890 | 0.273 |
| RS | 60 | 0 | 60 | 35.274 | 67.974 | 54.811 | 8.545 |
| TL | 60 | 0 | 60 | 0.000 | 14.000 | 6.945 | 4.754 |
| hardness | 60 | 0 | 60 | 108.000 | 567.000 | 255.483 | 141.500 |

(b) Correlation matrix (Pearson)

| Variables | pH | salinity | moisture | DPPH | TPC | TVC | LAB | RS | TL | hardness |
| --- | --- | --- | --- | --- | --- | --- | --- | --- | --- | --- |
| pH | **1.00** | -0.25 | 0.06 | **0.69** | **0.78** | **0.33** | **0.65** | **-0.32** | **-0.49** | **0.50** |
| salinity | -0.25 | **1.00** | **0.57** | -0.07 | -0.14 | **-0.31** | -0.19 | **-0.38** | **0.29** | -0.09 |
| moisture | 0.06 | **0.57** | **1.00** | 0.13 | -0.09 | -0.25 | -0.02 | **-0.87** | 0.23 | 0.07 |
| DPPH^*^ | **0.69** | -0.07 | 0.13 | **1.00** | **0.75** | **0.31** | **0.94** | **-0.50** | **-0.81** | **0.88** |
| TPC | **0.78** | -0.14 | -0.09 | **0.75** | **1.00** | **0.31** | **0.74** | -0.22 | **-0.64** | **0.56** |
| TVC | **0.33** | **-0.31** | -0.25 | **0.31** | **0.31** | **1.00** | **0.34** | -0.01 | **-0.33** | **0.32** |
| LAB | **0.65** | -0.19 | -0.02 | **0.94** | **0.74** | **0.34** | **1.00** | **-0.37** | **-0.93** | **0.93** |
| RS | **-0.32** | **-0.38** | **-0.87** | **-0.50** | -0.22 | -0.01 | **-0.37** | **1.00** | 0.14 | **-0.42** |
| TL | **-0.49** | **0.29** | 0.23 | **-0.81** | **-0.64** | **-0.33** | **-0.93** | 0.14 | **1.00** | **-0.91** |
| hardness | **0.50** | -0.09 | 0.07 | **0.88** | **0.56** | **0.32** | **0.93** | **-0.42** | **-0.91** | **1.00** |

^*^DPPH, DPPH radical scavenging activity; LAB, lactic acid bacteria; RS, reducing sugar; TL, thawing loss; TPC, total phenolic content; TVB, Total viable bacteria

(c) p-value (Pearson)

| Variables | pH | salinity | moisture | DPPH | TPC | TVC | LAB | RS | TL | hardness |
| --- | --- | --- | --- | --- | --- | --- | --- | --- | --- | --- |
| pH | **0** | 0.056 | 0.637 | **<0.0001** | **<0.0001** | **0.011** | **<0.0001** | **0.013** | **<0.0001** | **<0.0001** |
| salinity | 0.056 | **0** | **<0.0001** | 0.577 | 0.281 | **0.017** | 0.156 | **0.003** | **0.024** | 0.490 |
| moisture | 0.637 | **<0.0001** | **0** | 0.310 | 0.491 | 0.054 | 0.897 | **<0.0001** | 0.077 | 0.584 |
| DPPH | **<0.0001** | 0.577 | 0.310 | **0** | **<0.0001** | **0.015** | **<0.0001** | **<0.0001** | **<0.0001** | **<0.0001** |
| TPC | **<0.0001** | 0.281 | 0.491 | **<0.0001** | **0** | **0.016** | **<0.0001** | 0.088 | **<0.0001** | **<0.0001** |
| TVC | **0.011** | **0.017** | 0.054 | **0.015** | **0.016** | **0** | **0.008** | 0.938 | **0.011** | **0.013** |
| LAB | **<0.0001** | 0.156 | 0.897 | **<0.0001** | **<0.0001** | **0.008** | **0** | **0.003** | **<0.0001** | **<0.0001** |
| RS | **0.013** | **0.003** | **<0.0001** | **<0.0001** | 0.088 | 0.938 | **0.003** | **0** | 0.290 | **0.001** |
| TL | **<0.0001** | **0.024** | 0.077 | **<0.0001** | **<0.0001** | **0.011** | **<0.0001** | 0.290 | **0** | **<0.0001** |
| Tex | **<0.0001** | 0.490 | 0.584 | **<0.0001** | **<0.0001** | **0.013** | **<0.0001** | **0.001** | **<0.0001** | **0** |

**Table S2.** Principal component analysis (PCA) results for frozen kimchi quality parameters.
(a) Eigenvalues, variance explained, and cumulative variance for the principal components.

|  | PC1 | PC2 | PC3 | PC4 | PC5 | PC6 | PC7 | PC8 | PC9 | PC10 |
| --- | --- | --- | --- | --- | --- | --- | --- | --- | --- | --- |
| Eigenvalue | 5.117 | 2.389 | 0.862 | 0.737 | 0.557 | 0.167 | 0.086 | 0.034 | 0.030 | 0.022 |
| Variability (%) | 51.168 | 23.886 | 8.618 | 7.371 | 5.569 | 1.672 | 0.857 | 0.344 | 0.299 | 0.217 |
| Cumulative % | 51.168 | 75.053 | 83.671 | 91.042 | 96.611 | 98.283 | 99.140 | 99.484 | 99.783 | 100.000 |

(b) Loadings of each variable (TL, hardness, LAB, DPPH, TPC, RS, TVB) on PC1 and PC2.

|  | PC1 | PC2 | PC3 | PC4 | PC5 |
| --- | --- | --- | --- | --- | --- |
| pH | 0.763 | -0.005 | 0.448 | -0.380 | -0.028 |
| salinity | -0.209 | 0.743 | -0.187 | 0.029 | 0.598 |
| moisture | 0.020 | 0.958 | 0.158 | 0.054 | -0.181 |
| DPPH | 0.956 | 0.147 | -0.074 | -0.014 | 0.008 |
| TPC | 0.815 | -0.082 | 0.195 | -0.381 | 0.273 |
| Total viable bacteria | 0.430 | -0.354 | 0.523 | 0.610 | 0.210 |
| LAB | 0.972 | -0.011 | -0.183 | 0.029 | -0.017 |
| RS | -0.417 | -0.842 | -0.175 | -0.134 | 0.208 |
| Thawing loss | -0.888 | 0.221 | 0.354 | -0.087 | 0.018 |
| Hardness | 0.905 | 0.082 | -0.308 | 0.215 | -0.056 |

(c) PCA scores of each treatment group (SG, GG, GC, GG-CX, GC-CX) on PC1 and PC2

| Samples^1)^ | F1 | F2 | F3 | F4 | F5 |
| --- | --- | --- | --- | --- | --- |
| SG_0m | 3.434 | 2.508 | -0.291 | 0.425 | -1.130 |
| \| SG_0m \| \| --- \| | 3.615 | 1.722 | 0.396 | 1.634 | -0.393 |
| SG_0m | 3.255 | 2.325 | -0.777 | 0.611 | -1.015 |
| SG_1m | -0.221 | 2.135 | 1.924 | -0.082 | 0.687 |
| SG _1m | -0.198 | 2.129 | 1.588 | -0.673 | 0.167 |
| SG _1m | -0.433 | 1.797 | 1.151 | -0.727 | 0.021 |
| SG _2m | -1.597 | 1.852 | 0.967 | -0.666 | -0.346 |
| SG _2m | -2.021 | 1.518 | 1.312 | 0.284 | 0.135 |
| SG _2m | -1.609 | 2.028 | 1.251 | -0.107 | 0.206 |
| SG _3m | -3.152 | 1.152 | 0.289 | 0.929 | -1.865 |
| SG _3m | -3.031 | 0.957 | 0.725 | 1.301 | -1.698 |
| SG _3m | -3.447 | 1.559 | -0.651 | -0.083 | -2.167 |
| GG_0m | 3.302 | 1.325 | -0.765 | 0.201 | 0.138 |
| GG_0m | 3.445 | 1.656 | -1.339 | -0.372 | -0.019 |
| GG_0m | 3.092 | 1.074 | -1.027 | 0.165 | 0.198 |
| GG_1m | -0.127 | 0.846 | 2.056 | 0.206 | 0.058 |
| GG_1m | -0.361 | 0.191 | 1.792 | -0.530 | -0.441 |
| GG_1m | -0.437 | 0.780 | 0.426 | -0.984 | -0.777 |
| GG_2m | -1.981 | 0.535 | 0.196 | 0.043 | 0.501 |
| GG_2m | -1.792 | 0.665 | -0.012 | -0.614 | 0.061 |
| GG_2m | -1.790 | 0.911 | 0.296 | -0.269 | 1.224 |
| GG_3m | -2.569 | 0.029 | -0.174 | 1.252 | 0.365 |
| GG_3m | -3.241 | 0.146 | -0.885 | 0.380 | 0.060 |
| GG_3m | -2.992 | 0.390 | -1.422 | 0.038 | 0.038 |
| GC_0m | 4.128 | -1.279 | 0.930 | -0.015 | -0.420 |
| GC _0m | 3.817 | -0.837 | -0.311 | -0.986 | -0.838 |
| GC _0m | 3.797 | -0.930 | 0.398 | -0.314 | -0.413 |
| GC _1m | 1.410 | -1.507 | 1.292 | -0.483 | 0.415 |
| GC _1m | 1.279 | -1.400 | 1.342 | -0.894 | -0.151 |
| GC _1m | 1.089 | -1.663 | 0.766 | -0.691 | -0.018 |
| GC _2m | -0.535 | -1.666 | 0.291 | -0.516 | 0.530 |
| GC _2m | -0.770 | -1.461 | -0.388 | -1.784 | 0.028 |
| GC _2m | -0.889 | -1.800 | -0.319 | -1.036 | -0.277 |
| GC _3m | -1.074 | -2.307 | 0.655 | 1.090 | -0.080 |
| GC _3m | -1.290 | -2.216 | 0.347 | 0.524 | -0.144 |
| GC _3m | -1.333 | -2.302 | -0.299 | 0.412 | -0.028 |
| GG-CX_0m | 2.706 | 1.383 | -1.423 | 0.239 | 0.690 |
| GG-CX_0m | 2.175 | 1.578 | -1.977 | -0.977 | 0.254 |
| GG-CX_0m | 2.292 | 1.504 | -1.520 | 0.271 | 0.523 |
| GG-CX_1m | -0.644 | 1.556 | 0.034 | -0.624 | 0.619 |
| GG-CX_1m | -0.701 | 1.331 | -0.397 | -0.599 | 0.061 |
| GG-CX_1m | -0.828 | 1.341 | -0.434 | -0.896 | 0.285 |
| GG-CX_2m | -1.633 | 0.518 | -0.209 | -0.036 | 1.269 |
| GG-CX_2m | -1.510 | 0.608 | -0.440 | -0.083 | 1.150 |
| GG-CX_2m | -1.539 | 0.381 | -0.381 | 0.441 | 1.200 |
| GG-CX_3m | -2.467 | 0.046 | -0.424 | 1.721 | 0.586 |
| GG-CX_3m | -2.588 | 0.235 | -0.987 | 1.416 | 0.570 |
| GG-CX_3m | -2.962 | 0.442 | -1.467 | 0.465 | 0.260 |
| GC-CX_0m | 4.382 | -1.477 | 0.796 | 2.146 | 0.456 |
| GC -CX_0m | 3.693 | -0.917 | -0.012 | 0.804 | 0.098 |
| GC -CX_0m | 3.905 | -1.277 | 0.044 | 1.400 | 0.183 |
| GC -CX_1m | 0.834 | -1.152 | -0.169 | -1.703 | -0.064 |
| GC -CX_1m | 0.602 | -1.239 | -0.723 | -1.728 | -0.818 |
| GC -CX_1m | 1.085 | -1.682 | 1.030 | -0.139 | -0.035 |
| GC -CX_2m | -0.373 | -1.858 | -0.259 | 0.102 | 1.403 |
| GC -CX_2m | -0.591 | -1.625 | -0.581 | -0.736 | 0.676 |
| GC -CX_2m | -0.489 | -1.907 | 0.100 | -0.268 | 1.195 |
| GC -CX_3m | -0.869 | -3.386 | 0.071 | 1.582 | -0.623 |
| GC -CX_3m | -1.566 | -2.850 | -1.048 | 0.025 | -1.200 |
| GC -CX_3m | -1.688 | -2.413 | -1.358 | -0.494 | -1.352 |

^1)^ SG, general dehydration with salt; GG, general dehydration with glucose; GC, centrifugal dehydration with glucose; GG-CX, general dehydration with glucose and mixture of CMC and xanthan gum; GC-CX, centrifugal dehydration with glucose and mixture of CMC and xanthan gum.
